# OTUB1 Promotes Progression and Proliferation of Prostate Cancer via Deubiquitinating and Stabling Cyclin E1

**DOI:** 10.3389/fcell.2020.617758

**Published:** 2021-01-18

**Authors:** Yihao Liao, Ning Wu, Keke Wang, Miaomiao Wang, Youzhi Wang, Jie Gao, Boqiang Zhong, Fuling Ma, Yudong Wu, Ning Jiang

**Affiliations:** ^1^Tianjin Institute of Urology. The Second Hospital of Tianjin Medical University, Tianjin Medical University, Tianjin, China; ^2^Key Laboratory of Breast Cancer Prevention and Therapy, State Ministry of Education, National Clinical Research Center for Cancer, Tianjin Medical University Cancer Hospital and Institute, Tianjin, China; ^3^Key Laboratory of Cancer Prevention and Therapy, Tianjin Clinical Research Center for Cancer, Tianjin Medical University Cancer Hospital and Institute, Tianjin, China; ^4^Department of Urology, The First Affiliated Hospital of Zhengzhou University, Zhengzhou, China

**Keywords:** cyclin E1, OTUB1, prostate cance, progression, proliferation

## Abstract

**Background:** Prostate cancer (PCa) is currently the most common cancer among males worldwide. It has been reported that OTUB1 plays a critical role in a variety of tumors and is strongly related to tumor proliferation, migration, and clinical prognosis. The aim of this research is to investigate the regulatory effect of OTUB1 on PCa proliferation and the underlying mechanism.

**Methods:** Using the TCGA database, we identified that OTUB1 was up-regulated in PCa, and observed severe functional changes in PC3 and C4-2 cells through overexpression or knock down OTUB1. Heterotopic tumors were implanted subcutaneously in nude mice and IHC staining was performed on tumor tissues. The relationship between OTUB1 and cyclin E1 was identified via Western blotting and immunoprecipitations assays.

**Results:** We found that the expression of OTUB1 in PCa was significantly higher than that in Benign Prostatic Hyperplasia (BPH). Overexpression OTUB1 obviously promoted the proliferation and migration of PC3 and C4-2 cells via mediating the deubiquitinated Cyclin E1, while OTUB1 knockout has the opposite effect. The nude mice experiment further explained the above conclusions. We finally determined that OTUB1 promotes the proliferation and progression of PCa via deubiquitinating and stabling Cyclin E1.

**Conclusions:** Our findings reveal the critical role of OTUB1 in PCa, and OTUB1 promotes the proliferation and progression of PCa via deubiquitinating and stabilizing Cyclin E1. Blocking OTUB1/Cyclin E1 axis or applying RO-3306 could significantly repress the occurrence and development of PCa. OTUB1/Cyclin E1 axis might provide a new and potential therapeutic target for PCa.

## Introduction

Prostate cancer (PCa) is the most common malignant tumor in the United States. The prevalence of PCa approximately accounts for 20% of all types of cancers. In 2019, there were 174,650 new cases and 31,620 deaths from PCa (Siegel et al., 2019). If detected early and treated aggressively, the 5-year survival rate of PCa will almost reach 100%. However, many patients with PCa are diagnosed in the late stage, and their survival rate declines drastically because PCa has no obvious symptoms except urinary tract infection (Nguyen-Nielsen and Borre, 2016). Due to the irreplaceable role of androgen receptor (AR) in the development of PCa, the most important and standard treatment is androgen deprivation therapy (ADT) (Murillo-Garzón and Kypta, 2017; Bastos and Antonarakis, 2018). ADT mainly includes drug castration and surgical castration, but most patients eventually develop to castration-resistant prostate cancer (CRPC), even metastasis castration resistant prostate cancer (mCRPC), without effective treatment (Gasnier and Parvizi, 2017; Hossain et al., 2018). CRPC and mCRPC still are the most difficult problems during the diagnosis and treatment of prostate cancer. In recent years, with the development of urology, chemotherapy, radiotherapy, target therapy, and immunotherapy have emerged, and the overall survival rate has been prominently improved (Sebesta and Anderson, 2017; Altwaijry et al., 2018; Komura et al., 2018). At present, there is still a lack of effective and sensitive drugs for prostate cancer, especially the urgent demand for new drugs to treat CRPC (Smolle et al., 2017).

Ubiquitination is a vital pathway for protein degradation and conducts a crucial regulatory factor in many cellular signal pathways (Popovic et al., 2014). As a member of the deproteinized cysteine protease subfamily of the ovarian tumor domain (OTU) (Sivakumar et al., 2020), OTUB1 could stabilize the expression level of target protein and maintain its function by inhibiting ubiquitination degradation (Wiener et al., 2012). Many researchers have demonstrated that OTUB1 regulates lots of important cellular processes, such as DNA-reparation, cell signaling transduction, proliferation, and apoptosis (Nakada et al., 2010; Liu et al., 2019). Furthermore, OTUB1 plays an increasingly important and irreplaceable role in the field of cancer. For example, OTUB1 is found to be up-regulated in colorectal cancer (Zhou et al., 2014), gastric adenocarcinoma (Weng et al., 2016), esophageal cancer (Sun et al., 2020), ovarian cancer (Wang et al., 2016), human glioma (Xu et al., 2017), and hepatocellular carcinoma (Ni et al., 2017), which could promote tumor invasion and predict a poor prognosis. OTUB1 promotes tumor progression in two ways: to stabilize the expression of oncogenic genes by inhibiting the ubiquitination of target protein, and the other mode does not depend on the deproteinization manner (Saldana et al., 2019) but directly interacts with E2 ubiquitin ligase. These results imply that OTUB1 might provide a tumor associated biomarker and candidate target for PCa treatment. Currently, the relationship between OTUB1 and PCa has been preliminarily researched. Previous research verified that OTUB1 promotes prostate cancer invasion *in vitro* and aggravates tumorigenesis *in vivo* via regulating RhoA activity and p53 expression (Iglesias-Gato et al., 2015). The cyclin/Cdk complexes involved in cell cycle are the primary regulators during the various stages of mitosis, which could influence the conversion within different cell phases through the phosphorylation of cell phase-specific substrate proteins (Malumbres and Barbacid, 2009; Wei et al., 2020). Cyclin E1 is known to be a conserved protein and its essential function is to promote G1/S conversion. In previous studies, Cyclin E1/Cdk2 axis has been associated with the proliferation of various cancers in previous studies (Geng et al., 2003; Masaki et al., 2003). The anticancer effect of Cyclin E1/Cdk2 complexes has been extensively concerned in a variety of tumors, including ovarian cancer (Kanska et al., 2016), liver cancer (Bisteau et al., 2014; Ehedego et al., 2018), and so on (Fang et al., 2016).

In this study, we focus on the characteristics of OTUB1 involved in the process of cell cycle, and we investigate the specific mechanism of OTUB1 promoting tumor progression. Further experiments were performed to explore the possibility OTUB1 serves as a potential therapeutic target and diagnostic biomarker for PCa.

## Methods

### Clinical Samples

Clinical tissue samples were acquired from patients undergoing transurethral resection of the prostate in the Second Affiliated Hospital of Tianjin Medical University (Tianjin, China) and examined by a professional pathologist in order to obtain Gleason grade. This study was approved by the Ethics Committee of the Tianjin Medical University and strictly complied with the Helsinki Declaration of Human Rights.

### Prostate Cancer Cell Lines

Human prostate cancer cell lines (PC3 and C4-2) were obtained from ATCC cell bank. The cells were cultured with RPMI 1640 medium supplemented with 10% fetal bovine serum and 1% penicillin/streptomycin in a humidified environment containing 5% CO2 at 37°C.

### Cell Transfection and Inhibitor

A total of 6 × 10^5^ PC3 and C4-2 cells were seeded in 6-well plates. After 24 h, the cells were transfected with 2 μg plasmid or 100-nM siRNA with LipofectamineTM 2000 (Invitrogen) according to the manufacturer's protocol. The FLAG-OTUB1 plasmid and pcDNA3.1-OTUB1C91S plasmid was transfected into PC3 cell and C42 cell. The knockdown of OTUB1 and Cyclin E1 was generated by transient transfecting with RNA (OTUB1 siRNA and Cyclin E1 siRNA). After 48 h, the cells were collected for western blotting, MTT, transwell, and migration assays. The relative siRNA primers are showed in Supplementary Table 1. The cell cycle inhibitor RO-3306 was purchased from MCE, and different doses of RO-3306 were added into the 6-well plate respectively.

### Immunoprecipitation and Western Blot Analysis

Total protein was extracted from PC3, C4-2 cell lines, and tumor tissues using RIPA (Biosharp) and PMSF, and the BCA kit was used to determine the protein concentration. In the 10% acrylamide gels, an equal amount of protein sample (40 μg per channel) was separated by SDS-polyacrylamide gel electrophoresis (PAGE) and transferred to the poly vinylidine difluoride (PVDF) membrane (Millipore, Billerica, MA). The membrane was blocked in 5% fat-free milk and incubated overnight with the following primary antibodies: rabbit anti-OTUB1 (1:1,000 dilution; affinity), rabbit anti-GAPDH (1:1,000 dilution; Abcam), mouse anti-β-actin (1:1,000 dilution; CST), rabbit anti-Cyclin E1 (1:1,000 dilution; CST), and rabbit anti-FLAG (1:1,000 dilution; SIGMA) at 4°C. Then, the PVDF membrane were washed and incubated with anti-rabbit or anti-mouse IgG for 1 h at room temperature. The immunoreactive bands were detected by chemiluminescence methods and visualized using Luminescent Imaging Workstation, and the relative intensity was measured and analyzed using ImageJ software.

### Immunohistochemical Staining

The clinical tissue samples were collected from prostate surgery and the tumors of null mice were collected and preserved in formalin. The specimens were frozen, embedded in paraffin, and cut into 5 μm sections. The tissue sections were roasted at 65°C for 45 min, next de-waxed in xylene and rehydrated in graded alcohol. Citric acid buffer solution (pH adjusted to 6.0) was used for antigen recovery, under high fire for 5 min and middle-low fire for 10 min in turn. Endogenous peroxidase was blocked in 0.3% hydrogen peroxide and 1.5% horse serum for 10 min. Then the tissue sections were incubated with primary antibody (anti-OTUB1, 1:100 from affinity; anti-Cyclin E1 1:100 from affinity; Ki67 1:100 from Abcam) overnight at 4°C. After using rabbit/mouse universal secondary antibody IgG (1 h), the secondary antibody was detected with the Ultraview DAB detection kit (Zhongshan Co, China). The nuclei were stained with hematoxylin, then dehydrated and transparent, and the slides were sealed with neutral glue. The expression levels of OTUB1, ki-67, and Cyclin E1 were observed under Zeiss microscope (×200).

### Wound Healing Assay

PC3 and C4-2 cells were seeded on 6-well plate and grew to the pavement overnight. After 24 hours of transfection, a channel was drawn on the monolayer cells with 10 μL micropipette tip. Then PC3 and C4-2 cells were washed with PBS twice and cultured in 10% FBS 1640 at 5% CO_2_, 37°C for an additional 24 h. Photographs were taken by an inverted Leica phase contrast microscope at 0 h and 24 h.

### Clone Formation Assay

PC3 and C4-2 cells were digested and 2.0 × 10^3^ cells in each group were seeded into 6-well plate. After 24 h, OTUB1 siRNA, Cyclin E1 siRNA, negative control siRNA, OTUB1-overexpression, and otub1 c91s were transfected, respectively. The cells were cultured for 1 week. After washing with phosphate-buffered saline (PBS) buffer twice, 4% paraformaldehyde was used to fixate for 20 min. Then, an appropriate amount of crystal violet solution was added and stained for 30 min. After washing with PBS again and air drying, the software Image J was used for clones counting.

### MTT Assay

After 48 h of transfection, 2.0 × 10^3^ cells per well were seeded into 96-well plates and cultured at 37°C for 24 h, 48 h, 72 h, and 96 h. Then 30 uL MTT solution was added into each well at the indicated time, and cells were cultured for another 2 h at 37°C. Subsequently, the MTT solution was removed and 150 uL dimethyl sulfoxide (DMSO) was added into each well to dissolve formazan crystals. The absorbance was measured with a microplate reader at 490 nm.

### Transwell Migration Assay

PC3 and C4-2 cells were transfected with OTUB1-siRNA or negative-control siRNA and pcDNA3.1-OTUB1 plasmid respectively, which were suspended in 1,640 containing 10% FBS, and 2 × 104 cells were added to the top chamber of 24-well transwell plates (Corning, 8 m pore size), and 1,640 containing 10% FBS was added to the bottom chamber. After incubating at 37°C for 48 h, the chambers were washed with PBS twice, and these cells which migrated to the bottom chambers were fixed with paraformaldehyde and stained with crystal violet. Then the number of transitional cells in all chambers was calculated in the 5 visual fields.

### Animal Studies

Five-week-old male Babl/c mice (HFK Bio-Technology Co. Ltd, Beijing) were injected subcutaneously with 2 × 10^6^ PC3 cells with control, otub1, and otub1-c91s groups suspended in 0.1 mL of Matrigel (BD Biosciences) and 1,640. These cells were implanted subcutaneously into the dorsal flank on both sides of the mice. Once the diameter of tumors reached nearly 2 mm, the volume of tumor was measured daily for 10 days. These mice with overexpression otub1 were divided into two groups: one group was used as control group, and another were treated with RO-3306 4 mg/kg every 2 days via oral feeding. Tumor volume was recorded by digital caliper and the volume was estimated the formula 0.52^*^L^*^W^2^ (L = the length of tumor and W = the maximum width). At the 10th day, these mice were killed and tumors were extracted and measured. The tumors were fixated with paraformaldehyde, then immunohistochemistry staining was performed for OTUB1, Ki 67, and Cyclin E1. All procedures involving mice were approved by the University Committee on Use and Care of Animals at the Tianjin Medical University and met all regulatory standards.

## Results

### OTUB1 is Up-regulated in Prostate Cancer

**Figure 1:**
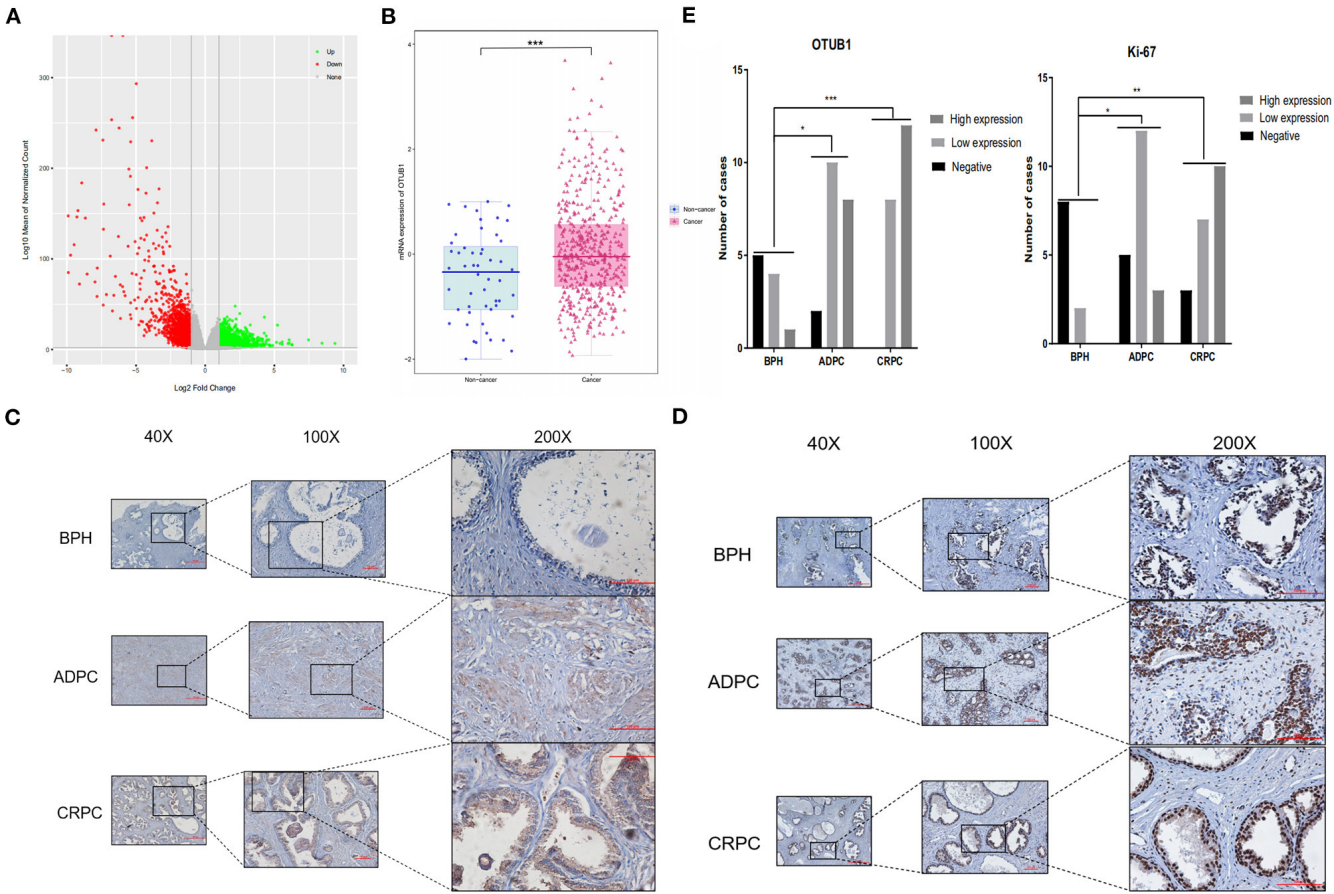
OTUB1 is up-regulated in prostate cancer. **(A)** The volcanic map depicting the dysfunctional genes in prostate cancer. **(B)** According to TCGA, OTUB1 is up-regulated in prostate cancer. **(C)** IHC staining showed that the expression of OTUB1 was up-regulated in human prostate cancer. **(D)** IHC staining showed that the expression of Ki-67 was up-regulated in human prostate cancer. **(E)** IHC of OTUB1 (*X*^2^ = 16.56; *P* = 0.0024) and Ki-67 (*X*^2^ = 20.2; *P* = 0.0005) was statistically analyzed according to the expression intensity. *P < 0.05; **P < 0.01; ***P < 0.001.

We filtered all dysregulated genes in prostate cancer profiles from TCGA database, identified 3,480 up-regulated genes and 2,592 down-regulated genes, and the results were presented in the volcano map (Figure 1A). Of the up-regulated genes, we observed that the expression of OTUB1 in PCa was higher than para-carcinoma tissue (Figure 1B). To further identify the deubiquitinating enzymes OTUB1 driving the prostate cancer progression, we conducted subsequent experiments and assays. In order to identify whether clinical data was consistent with the database, we collected clinical prostate cancer tissue and immunohistochemical staining was performed with OTUB1 (Figure 1C) and ki-67 antibody (Figure 1D). A Chi-square test was performed, and the results demonstrated that the expression of OTUB1 in PCa groups was higher than that in BPH group (*X*^2^ = 16.56; *P* = 0.0024), and the results of ki-67 were consistent with OTUB1 (*X*^2^ = 20.2; *P* = 0.0005). The detailed statistical results showed that the positive ratios of OTUB1 and Ki67 in ADPC and CRPC groups were higher than BPH group (Figure 1E).

### OTUB1 Promotes Proliferation and Invasion of PCa Cell

**Figure 2:**
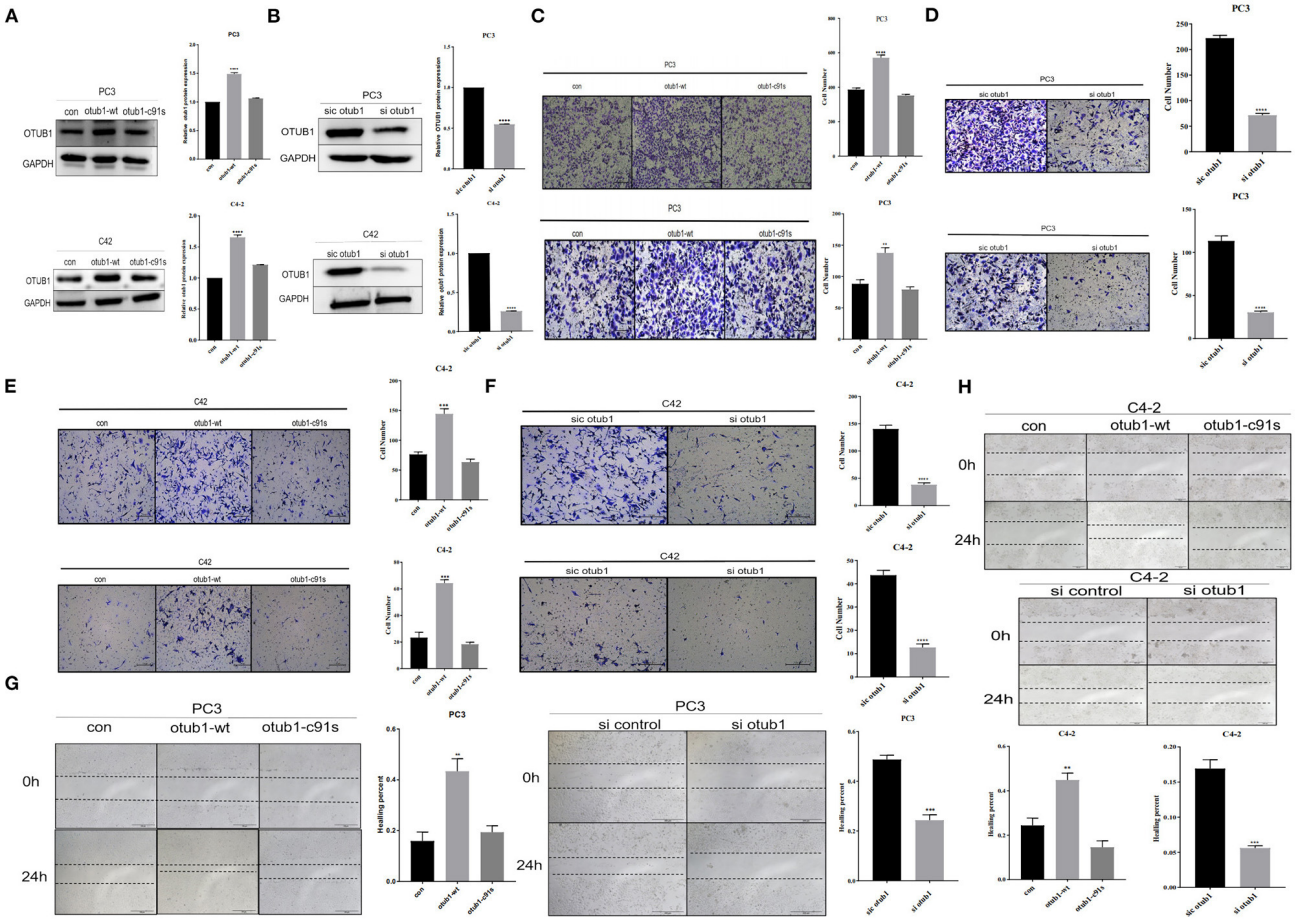
OTUB1 promotes the proliferation and invasion of PCa cells. **(A,B)** Western blotting detecting the expression of OTUB1 in C4-2 and PC3 cells transfected with overexpression OTUB1, otub1 c91s, and OTUB1 siRNA. **(C)** Transwell assay in PC3 cell transfected with overexpression OTUB1 and otub1 c91s. **(D)** Transwell assay in PC3 cell transfected with OTUB1 siRNA. **(E)** Transwell assay in C4-2 cell transfected with OTUB1 overexpression and otub1 c91s. **(F)** Transwell assay in C4-2 cell transfected with OTUB1 siRNA. **(G,H)** Wound healing assay in PC3 and C4-2 cells transfected with OTUB1 overexpression, otub1 c91s, and OTUB1 siRNA. *P < 0.05; **P < 0.01; ****P < 0.001.

We transfected PC3 and C42 cells with OTUB1 overexpression and otub1 c91s, and the expression level of OTUB1 was detected by Western blotting. The results showed that, compared with the control group, the expression level of OTUB1 transfected with otub1-c91s group (cells introduced by the mutated OTUB1 fragment) was not significantly changed, but the expression level of OTUB1 transfected with OTUB1 overexpression was significantly increased (Figure 2A). The gray value of OTUB1 was detected by Image analysis software Image J (Figure 2A). For further explanation, we transfected PC3 and C4-2 cells with OTUB1 siRNA, and the expression level of OTUB1 was decreased distinctly compared with the control group (Figure 2B). Recent studies have shown that OTUB1 is highly expressed in invasive tumor cells and plays an important role in its proliferation and invasion (Zhou et al., 2014, 2019, 2020; Weng et al., 2016; Yuan et al., 2017; Sun et al., 2020). The results showed that the migration and invasion ability of PC3 and C4-2 cells was significantly enhanced in increased OTUB1 group compared with control group (Figures 2C,E). On the contrary, PC3 and C4-2 cells transfected with OTUB1 siRNA dramatically attenuated the migration and invasion ability (Figures 2D,F).

Cell healing assays is one of the methods to determine the movement characteristics of tumor cells. In order to eliminate the interference of cell proliferation and observe the migration ability of tumor cells, the scratch injury was applied to monolayer cells *in vitro*. The effect of OTUB1 expression level on the migration ability of prostate cancer PC3 and C4-2 cells was observed. The results showed that the migration of OTUB1 overexpression group was significantly improved compared with the control group (Figure 2G), but there was no significant difference between the otub1 c91s group and the control group. The difference was statistically significant (*P* < 0.01). The similar results were also found in PC3 and C4-2 cells transfected with OTUB1 siRNA, the migration ability of PC3 and C4-2 cells was dramatically attenuated (Figure 2H). The results showed that OTUB1 expression significantly influenced cells migration (Figures 2G,H).

### Effects of Increased OTUB1 Expression on PC3 and C4-2 Cells Cycle Distribution

**Figure 3:**
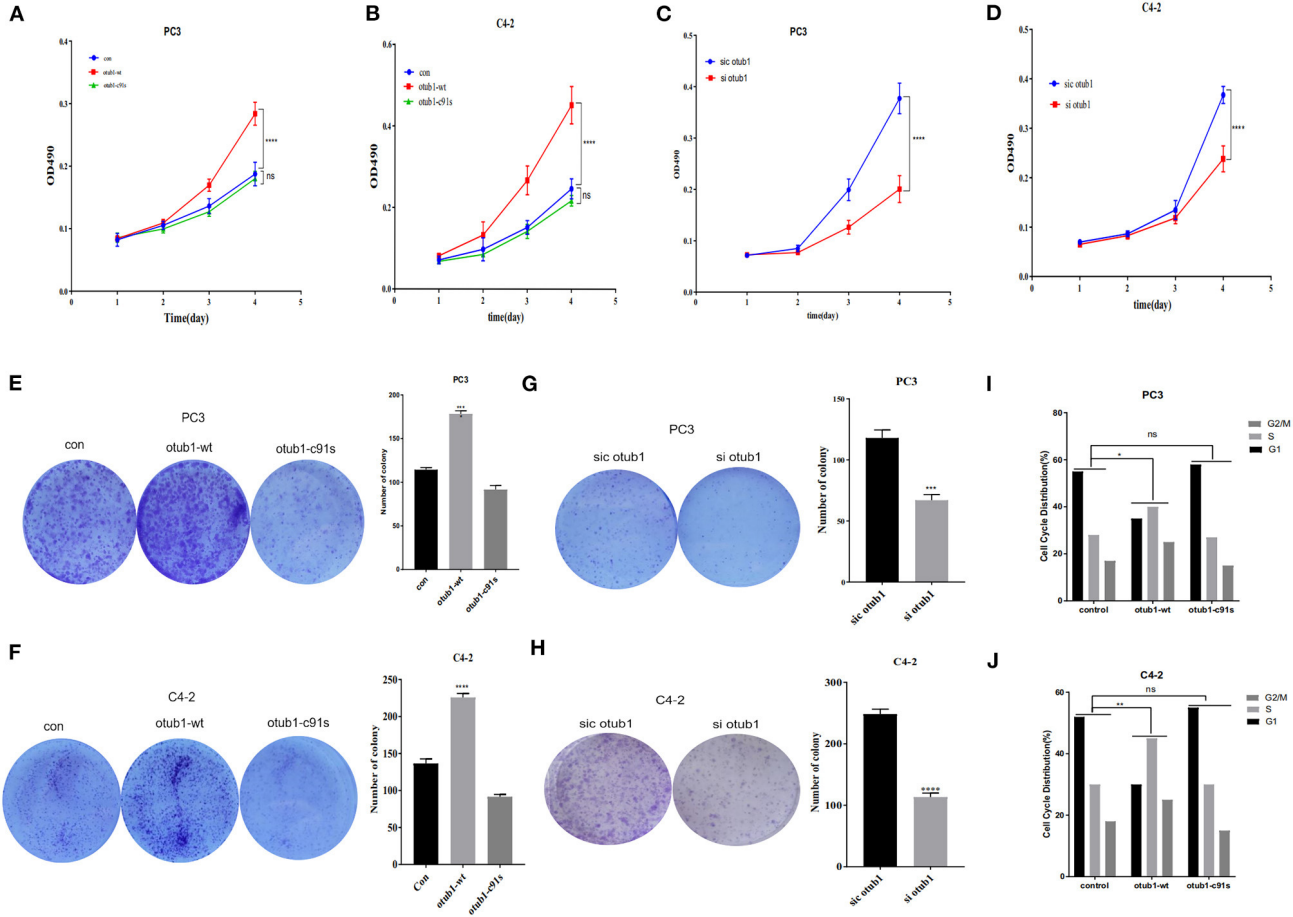
Effects of increased OTUB1 expression on the cells cycle distribution of PC3 and C4-2 cells. **(A,B)** MTT assays in PC3 and C4-2 cells transfected with otub1 overexpression and otub1 c91s. **(C,D)** MTT assays in PC3 and C4-2 cells transfected with OTUB1 siRNA. **(E,F)** Colony formation assays in PC3 and C4-2 cells transfected with OTUB1 overexpression and otub1 c91s. **(G,H)** Colony formation assays in PC3 an C4-2 cells transfected with OTUB1 siRNA. **(I,J)** Effects of increased otub1 expression on the cell cycle distribution of PC3 (*X*^2^ = 12.59; *P* = 0.0135) and C4-2 (*X*^2^ = 15.17; *P* = 0.0044) cells. *P < 0.05; **P < 0.01; ***P < 0.001; ****P < 0.0001.

The cell growth curve was drawn, and the proliferation absorbance of the control group, OTUB1 overexpression, and otub1 c91s at 24, 48, 72, and 96 h were detected, respectively. The results showed that the cells with increased OTUB1 expression had significantly higher growth ability than the control group, while the proliferation ability of otub1 c91s group had no significant change compared with the control group (*P* < 0.01; Figures 3A,B). In the same way, we transfected PC3 and C4-2 cells with OTUB1 siRNA and found the opposite results (Figures 3C,D). The results demonstrated that the expression of OTUB1 significantly affected the proliferation of PC3 and C4-2 cells (*P* < 0.01; Figures 3A–D). Clone formation rate is defined as the rate at which a single cell grows and forms small cell groups (clones). The cells were inoculated at a low density (2 × 10^3^cells /chamber). After 7 days of culture, all cells formed obvious colonies. The colony forming ability of PC3 and C4-2 cells was significantly enhanced in increased OTUB1 group (*P* < 0.01; Figures 3E,F). Similarly, we transfected PC3 and C4-2 cells with OTUB1 siRNA and the colony forming ability of PC3 and C4-2 cells was reduced significantly (*P* < 0.01; Figures 3G,H). Based on the above results, it could be seen that the expression levels of OTUB1 influence the ability of colony formation.

Cell cycle refers to the whole process from the end of the last mitosis to the completion of the next mitosis, including quiescent phase (G0), early DNA synthesis phase (G1), DNA synthesis phase (S), late DNA synthesis phase (G2), and division phase (M). After increasing the expression of OTUB1, the proliferation ability of prostate cancer cells was significantly enhanced (*P* < 0.01; Figures 3A–D). Cell cycle test results of PC3 and C4-2 showed that the ratio of G1 phase decreased in increased OTUB1 expression group, while the ratio of G2/M+S phase increased, the Chi-square test results of PC3 (*X*^2^ = 12.59; *P* = 0.0135) and C4-2 (*X*^2^ = 15.17; *P* = 0.0044) showed the statistical difference (Figures 3I,J). In conclusion, these experiments suggested that OTUB1 might influence the proliferation of PCa cells through altering the distribution of cell cycle (Figures 3I,J).

### OTUB1 Rescues Cyclin E1 From Proteasomal Degradation

**Figure 4:**
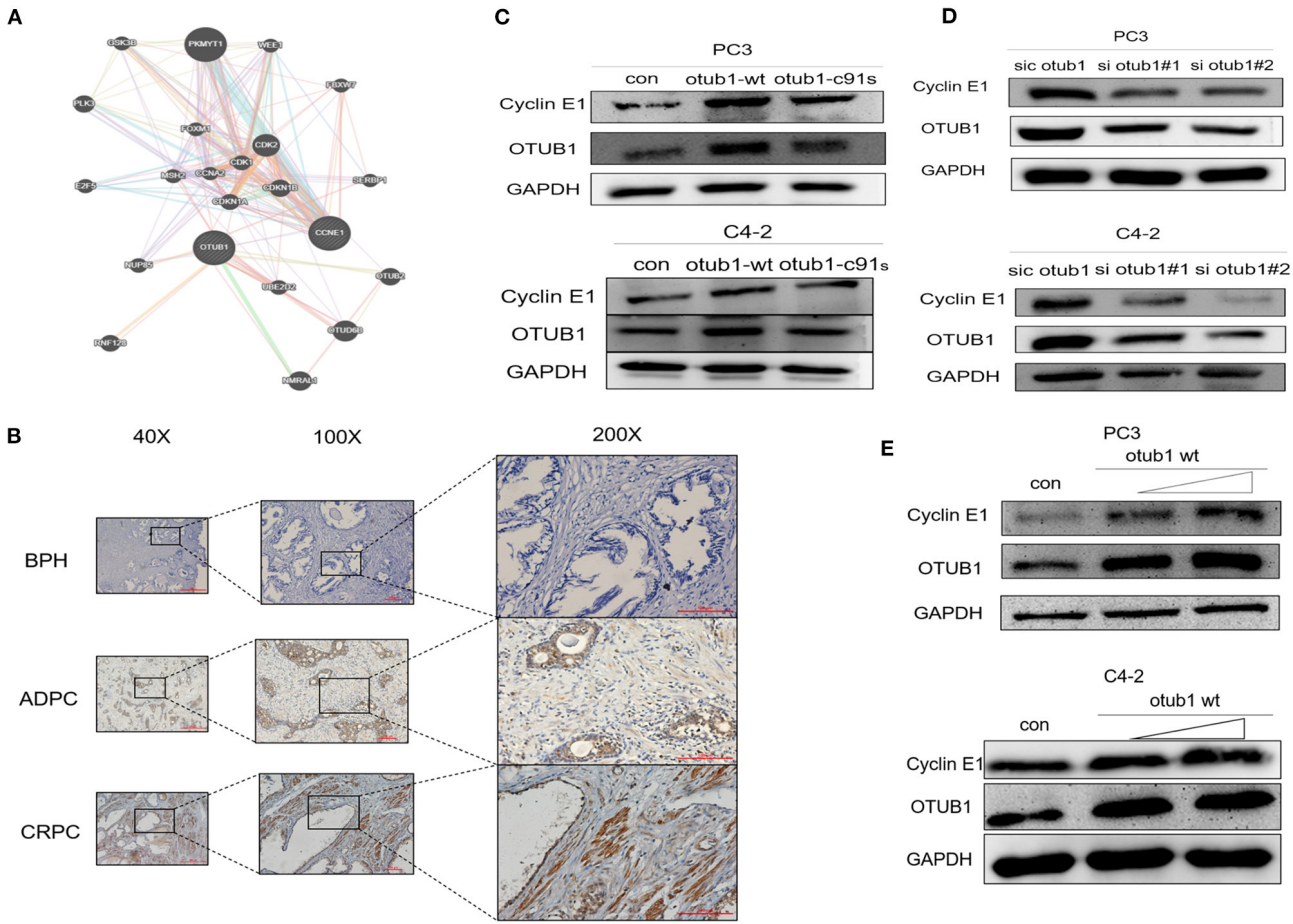
OTUB1 promotes and regulates the expression of Cyclin E1. **(A)** The relationship between OTUB1 and Cyclin E1 was analyzed via gene MANIA database. **(B)** The protein expression of Cyclin E1 in BPH, ADPC, and CRPC was detected by IHC staining. **(C)** Western blotting detecting the expression of Cyclin E1 in PC3 and C4-2 cells transfected with otub1 overexpression and otub1 c91s. **(D)** Western blotting detecting the expression of Cyclin E1 in PC3 and C4-2 cells transfected with OTUB1 siRNA. **(E)** Western blotting detecting the expression of Cyclin E1 in PC3 and C4-2 cells transfected with an increasing gradient OTUB1.

Next, we investigated how OTUB1 promotes the G1 cell cycle progression, and analyzed a series of OTUB1-related proteins through gene MANIA online database. Cyclin E1, a cell cycle-relative regulative key protein, was found to interact with OTUB1 closely (Figure 4A). To explore the relationship between OTUB1 and Cyclin E1, we further observed a consistent result with OTUB1 that the expression level of Cyclin E1 in ADPC and CRPC groups were significantly higher than that in BPH group via IHC assay (Figure 4B). To verify whether the function of Cyclin E1 is related to OTUB1, we observed that the expression of Cyclin E1 increased in increased OTUB1 expression group of PC3 and C4-2 cells (Figure 4C). In addition, we found a similar experiment result that the protein expression of OTUB1 and Cyclin E1 deceased obviously in PC3 and C4-2 cell transfected with OTUB1 siRNA (Figure 4D). The expression level of OTUB1 and Cyclin E1 was found to gradually increase with the gradient overexpression of OTUB1 in PC3 and C4-2 cells (Figure 4E). It could be inferred that up-regulated OTUB1 could promote the expression of Cyclin E1 from the above results, and we had sufficient evidence to predict that Cyclin E1 could interact with OTUB1.

**Figure 5:**
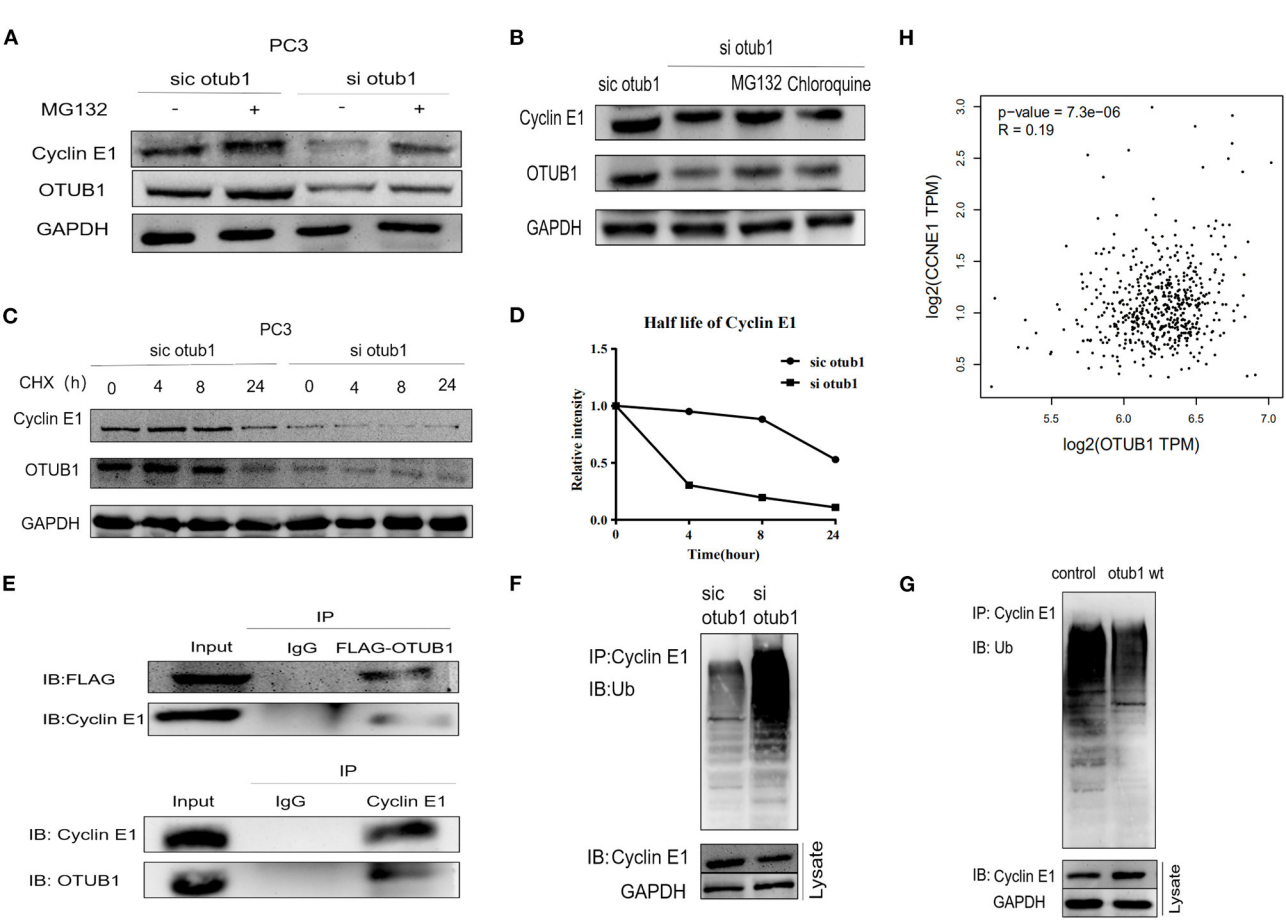
OTUB1 rescues Cyclin E1 from proteasomal degradation. **(A)** Western blotting detecting the expression of Cyclin E1 in PC3 cells transfected with OTUB1 siRNA and treated with 10 uM MG132 for 8 h. **(B)** Western blotting detecting the expression of Cyclin E1 in PC3 cells transfected with OTUB1 siRNA and treated with CHX and chloroquine for 8 h. **(C,D)** Western blotting detecting the expression of Cyclin E1 in PC3 cell transfected OTUB1 siRNA and treated with CHX for 0, 4, 8, and 24 h. **(E)** OTUB1 interacted with Cyclin E1 (upper panel) and Cyclin E1 interacted with OTUB1 (lower panel) were found via immunoprecipitation assay. **(F)** The effect of OTUB1 knockdown on ubiquitination of Cyclin E1 in PC3 cells. **(G)** The effect of increased OTUB1 on ubiquitination of Cyclin E1 in PC3 cells. **(H)** The correlation between OTUB1 and Cyclin E1 was analyzed online (*R* = 0.19, *P* < 0.01).

Further experiments are needed to verify this conclusion. Thus, we explored how OTUB1 influences the expression of Cyclin E1. PC3 cell transfected with OTUB1 siRNA were treated with DMSO or 10uM MG132 (a protease inhibitor) for 8 h, respectively. The results demonstrated that the expression of Cyclin E1 was decreased significantly in OTUB1 siRNA group compared with the control group, and MG132 could partially preserve the stability, indicating that OTUB1 influenced the expression of Cyclin E1 in a proteasome dependent manner (Figure 5A). Above results implied that Cyclin E1 might be regulated by OTUB1 via a deubiquitinating degradation manner. To identify this hypothesis, we treated PC3 cells transfected with OTUB1 siRNA with 10 uM MG132 and 200 uM chloroquine (a lysosomal enzyme inhibitor) for 8 h. We found that MG132 could partially maintain stability of Cyclin E1 in PC3 cell transfected with OTUB1 siRNA, while chloroquine could not (Figure 5B). To further explain the above results, PC3 cell transfected with OTUB1 siRNA were treated with 10 mg/ml cycloheximide (CHX), a protein synthesis inhibitor in eukaryotic cells, for 0, 4, 8, and 24 h, respectively. The results showed that CHX promoted the degradation of Cyclin E1 protein, and the decrease rate of Cyclin E1 was increased significantly in PC3 cell transfected with OTUB1 siRNA (Figures 5C,D). The results implied that Cyclin E1 was not a lysosomal enzyme degradation pathway but ubiquitin dependent degradation. The relationship between OTUB1 and Cyclin E1 was determined by immunoprecipitation. The results demonstrated that OTUB1 interacted with Cyclin E1, and Cyclin E1 was also linked with OTUB1 (Figure 5E). Another immunoprecipitation assay presented that knocking down the expression of OTUB1 could strengthen the degree of ubiquitination of Cyclin E1 (Figure 5F), while increased OTUB1 expression could weaken the ubiquitination of Cyclin E1 (Figure 5G). Therefore, these results demonstrated that OTUB1 could promote tumor proliferation and progression in prostate cancer via stabilizing the function and increasing the expression level of Cyclin E1. We analyzed the correlation between OTUB1 and Cyclin E1 via GEPIA online database, and the result presented that OTUB1 was positively correlated with Cyclin E1 (*R* = 0.19; *P* < 0.01; Figure 5H).

### OTUB1/Cyclin E1 Axis Promotes Prostate Cancer Cell Proliferation

**Figure 6:**
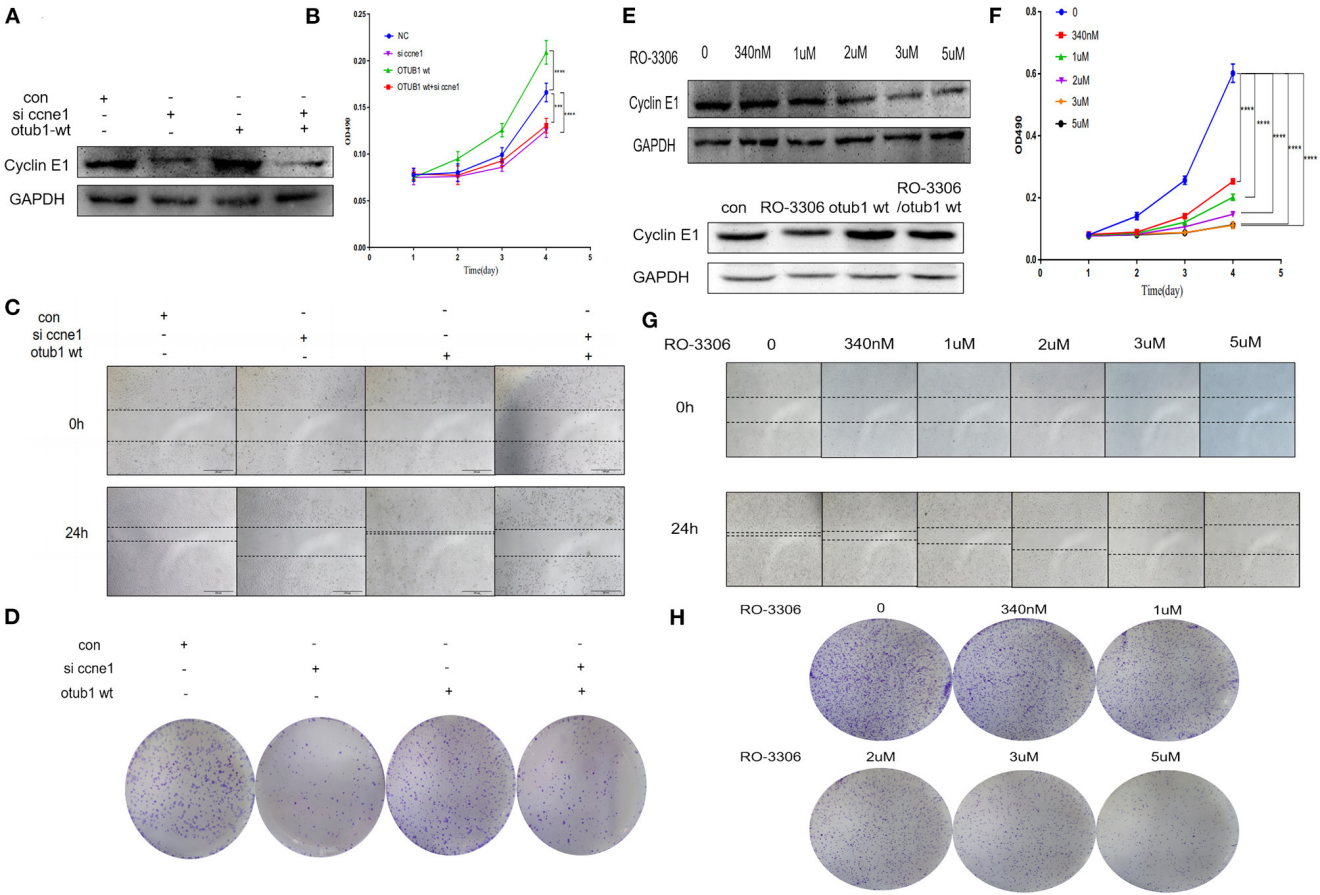
OTUB1/Cyclin E1 axis promotes the proliferation of PCa cells. **(A)** Western blotting detecting the expression of Cyclin E1 in PC3 cell transfected with OTUB1 overexpression and Cyclin E1 siRNA. **(B,D)** MTT assays and colony formation assays in PC3 cell transfected with OTUB1 overexpression and Cyclin E1 siRNA. **(C)** Transwell assay in PC3 cell transfected with OTUB1 overexpression and Cyclin E1 siRNA. **(E)** Western blotting detecting the expression of Cyclin E1 in PC3 cell treated with a different dose of RO-3306. **(F,H)** MTT assays and colony formation assays in PC3 cell treated with a different dose of RO-3306. **(G)** Transwell assay in PC3 cell treated with a different dose of RO-3306. ***P < 0.001; ****P < 0.0001.

To further explore the effect of OTUB1/Cyclin E1 axis upon PCa, PC3 cell were co-transfected with Cyclin E1 siRNA and OTUB1 overexpression. The expression of cyclin E1 was detected 48 h after transfection. The results demonstrated that OTUB1 could promote the expression of Cyclin E1, while Cyclin E1 expression was obviously decreased after transfection with Cyclin E1 siRNA. Interestingly, knockdown of Cyclin E1 attenuated the effect of OTUB1 overexpression (increasing the expression of Cyclin E1) (Figure 6A). MTT assay was used to detect cell proliferation, and the results demonstrated that Cyclin E1 knockdown obviously postponed cell proliferation, while the effect of OTUB1 overexpression promoting proliferation was restrained significantly to accompany with Cyclin E1 siRNA group (Figure 6B). These results were confirmed by healing assay and clone formation experiments, which were consistent with previous results (Figures 6C,D). These results demonstrated that OTUB1/Cyclin E1 axis promotes the proliferation and migration of prostate cancer. Interfering with the contact between OTUB1 and Cyclin E1 might provide a potential therapy for prostate cancer.

To identify the possibility of targeting prostate cancer with OTUB1/Cyclin E1 axis, we treated PC3 cell with RO-3306, a Cyclin E1/CDK2 related inhibitor, in the range dose of 0, 340 nM, 1 uM, 2 uM, 3 uM, and 5 uM. After treating with RO-3306 for 24 h, the protein was extracted and Western blotting was performed. We observed the phenomenon of the expression level of Cyclin E1 was degraded in a RO-3306 dose dependent manner. Interestingly, RO-3306 also attenuated the effect of OTUB1 overexpression on promoting Cyclin E1 (Figure 6E). The results were consistent with the knock down of Cyclin E1. More experiments were performed to further explore the effect of RO-3306 on cell proliferation and migration. MTT assays and clone formation assays demonstrated that the proliferation and migration ability declined gradually with the increase of RO-3306 concentration (Figures 6F,H). And the results of healing assays were coincided with those mentioned earlier results (Figure 6G). RO-3306 treatment significantly postponed the migration and proliferation ability of prostate cancer, and the clinical application of RO-3306 in the treatment of PCa might be a potential and effective measure, which could decrease the mortality of PCa patients.

### OTUB1 Promotes the Growth of PC3 Cell via Increasing Cyclin E1 *in vivo*

**Figure 7:**
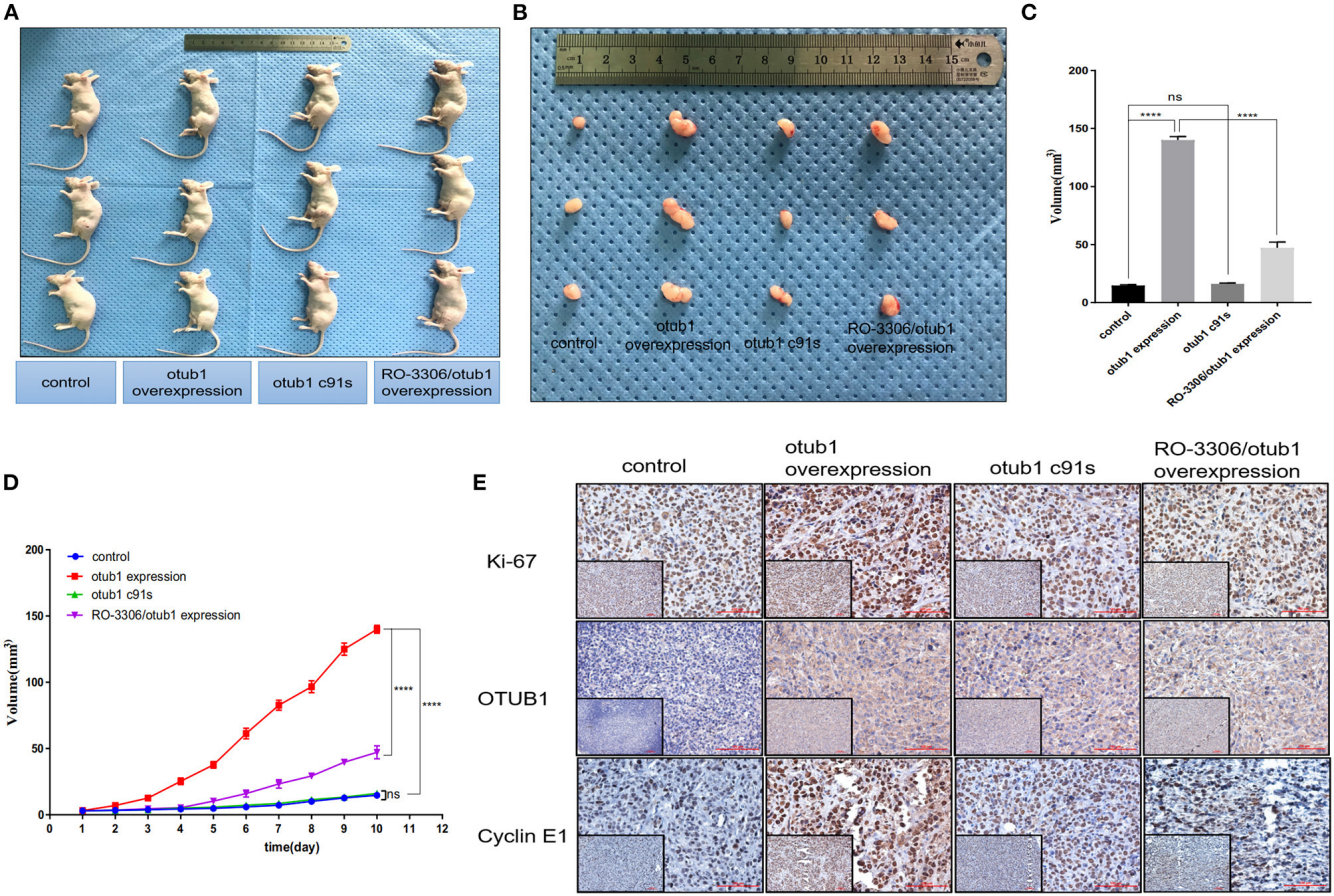
OTUB1 promotes the growth of PC3 cell via increasing Cyclin E1 *in vivo*. **(A,B)** PC3 cells transfected with OTUB1 overexpression and otub1 c91s were transplanted subcutaneously in nude mice. The effect of otub1 overexpression, otub1 c91s, and RO-3306 on the growth of PCa. **(C)** The tumor volume was measured with a caliper. **(D)** The tumor volumes were measured daily when the diameter grew into 2 mm. **(E)** IHC staining detecting the expression of OTUB1, ki-67, and Cyclin E1 in these tumors from nude mice. ****P < 0.0001.

Male nude mice aged 4–6 weeks were selected as animal models. We planted 2 × 10^6^ PC3 cells transfected with overexpressed OTUB1 and otub1 c91s in mouse groin, respectively. We began to measure the size of the tumors daily with a vernier caliper for 10 days, when the tumor grew to a diameter of 2 mm (Figure 7D). After 10 days, the animals were sacrificed with cervical dislocation, and the solid tumor was removed under sterile conditions. The tumor volume of OTUB1 overexpression group was distinctly different from that of the control group, but there was no obvious change in the otub1 c91s group compared with the control group (Figures 7A–C). We further found an interesting result that the tumor volume was restrained significantly compared with OTUB1 overexpression group, when these mice were oral administrated with RO-3306 in a dose of 4 mg/kg every day (Figures 7A–C). In addition, we found that tumor incidence in OTUB1 overexpressed group was significantly faster than that in the control group, while RO-3306 restrained the tumor incidence in the OTUB1 overexpression group (Figure 7D), indicating the critical role of OTUB1/Cyclin E1 axis in tumor formation. Immunohistochemical staining assay showed that the protein expression levels of OTUB1, Ki-67, and Cyclin E1 were higher in the tumors tissue with increased otub1 compared with the control group, and the expression of Cyclin E1 declined after the use of RO-3306, while the expression level of OTUB1 did not decrease (Figure 7E). The results further illustrated that OTUB1 regulated the expression and function of Cyclin E1 *in vivo*, and targeting OTUB1/Cyclin E1 axis might provide a potential therapeutic method for these patients with prostate cancer.

## Discussion

In our research, we found that the expression levels of OTUB1 are up-regulated in PCa, OTUB1 could promote the proliferation and progression of PCa via deubiquitinating and stabling the expression of Cyclin E1 protein. Many previous researches have shown that OTUB1 is frequently up-regulated in many cancers, such as esophageal squamous cell carcinoma and colon cancer (Liu et al., 2014). Based on these findings of previous studies, OTUB1 promotes the metastasis of esophageal squamous cell carcinoma by modulating snail stability (Zhou et al., 2018), while different expression of OTUB1 affects the migration and progression of colorectal cancer through the regulation of ERRα or mir-542-3p (Yuan et al., 2017; Zhou et al., 2019). OTUB1 not only regulates cancer metastasis but also chemoresistance. Karunarathna et al. found that OTUB1 inhibited the ubiquitination and degradation of FOXM1 in breast cancer, and mediated epirubicin resistance (Karunarathna et al., 2016). Recently, some researchers have proved that OTUB1 could attenuate interferon response to hepatitis B virus infection (Xie et al., 2020). The effects of OTUB1 in a variety of tumors reminds us that OTUB1 might play an important role during cancer evolution and some physiological activities.

In our research, we first found the expression of OTUB1 was up-regulated in PCa compared with normal prostate tissue from TCGA database. Next, we conducted immunohistochemical staining with PCa tissues and BPH to prove the above conclusion. The results were consistent with the database conclusion (Figure 1). To further explore the specific role of OTUB1 in the progression of prostate cancer, we performed a series of experiments to monitor the changes of migration ability by altering the expression of OTUB, and the results demonstrated that increased OTUB1 could significantly promote the migration and invasion ability of PC3 cell and C4-2 cells. When we knocked down the expression of OTUB1, the ability to influence migration and invasion was obviously decreased compared with untreated PC3 cell and C4-2 cells (Figure 2). Previous studies found the effect of OTUB1 on prostate cancer, which showed that there was no significant deviation in PCa proliferation between decreased OTUB1 and the control group (Iglesias-Gato et al., 2015). In this research, we found that increased OTUB1 could promote proliferation of PC3 and C4-2 cells. We also found that G1 phase of cell cycle was shortened with the elevated expression of OTUB1 (Figure 3). To investigate the effect of OTUB1 on cell proliferation, we found that increased OTUB1 could increase the expression of Cyclin E1. Further experiments presented that Cyclin E1 interacted with OTUB1. OTUB1 could mediate the deubiquitination of Cyclin E1 and stabilize the expression level and function of Cyclin E1. Compared with PC3 cells transfected with OTUB1 overexpression alone, the proliferation of PC3 cell was decreased when co-transfected with OTUB1 overexpression and Cyclin E1 siRNA (Figures 4–6). These results suggested that OTUB1 might promote the proliferation of PCa via mediating Cyclin E1. The experimental results *in vivo* were consistent with those of the above-mentioned cell results *in vitro* (Figure 7). So far, we have a lot of evidence to identify the hypothesis that OTUB1 promotes the progression and proliferation of prostate cancer via mediating and stabling Cyclin E1. Previous researches had proved that Cyclin E1 belongs to a highly conserved Cyclin family, and its members are characterized by a dramatic periodicity in protein abundance through the cell cycle (Masaki et al., 2003). Cyclins could function as the regulators of CDK kinases (Hu et al., 2014; Asghar et al., 2015). This cyclin forms a complex and functions as a regulatory subunit of CDK2, whose activity is required for G1/S transition of cell cycle (Xu et al., 2019). Many researches have presented that Cyclin E1 could mediate the progression of many tumors, such as hepatocellular carcinoma (Sonntag et al., 2018; Xu et al., 2019), ovarian cancer (Au-Yeung et al., 2017), and breast cancer (Turner et al., 2019). Previous researches have proved that Cyclin E1 has several functional domains, mainly including a central cyclin homology district (interacting with CDK2), a unique N-terminal region, and a C-terminal PEST sequence, which are often detected in protein degraded through the ubiquitin system (Lew et al., 1991; Richardson et al., 1993; Honda et al., 2005; Rath and Senapati, 2014). As an unstable protein, Cyclin E1 is degraded by two distinct pathways involving the ubiquitin-proteasome system mediated by Cul1 or Cul3, which belongs to the cullin-RING family of ubiquitin ligases (Welcker et al., 2003; Davidge et al., 2019). And Cul1 mediated-degradation requires phosphorylation of Cyclin E1 at T77 and T395 to produce ubiquitylation of cyclin E (Minella et al., 2008). Previous studies have shown that Cul3 degrades Cyclin E that was not bound to Cdk2 and regulation of Cyclin E by Cul3 (McEvoy et al., 2007; Davidge et al., 2019). Although our research found that the deubiquitinase OTUB1 promotes the stability of Cyclin E1 and the progression of prostate cancer, the specific mechanism of OTUB1 mediated function of Cyclin E1 remains to be further studied. The interaction among OTUB1, Cu11, and Cu13 might become the internal reason for the stable functions of Cyclin E1 and its subsequent functions. The next aim of our research focuses on the relationship between OTUB1 and ubiquitin ligases. Not only prostate cancer and the above-mentioned cancers, Kai Zhou et al. found that OTUB1 could promote the progression of renal cell carcinoma via mediating the deubiquitination of FOXM1 and up-regulating the expression of ECT-2 (K. Zhou et al., 2020). Other researchers have proved that SP1 regulates the progression of non-small-cell lung cancer by recruiting OTUB1 (Xie et al., 2019).

Based on the above results, we found that the high expression level of OTUB1 promoted the migration and invasion of PCa cells, while the low expression of OTUB1 decreased the migration and invasion of PCa cells. Previous studies on the biological function of OTUB1 are contradictory. The initial study found that OTUB1 attributed to the stability of P53 protein, and thus inhibiting cell proliferation. Recent studies have indicated that OTUB1 is involved in the invasion and migration of malignant tumors. However, in our study, we discovered that high level of OTUB1 promoted cell proliferation, while low expression of OTUB1 had the opposite effect. And we further found that OTUB1 promoted the progression of PCa via regulating the stability and interacting with Cyclin E1. Cyclin E1 was mainly degraded in a ubiquitination manner, yet OTUB1 inhibited the ubiquitination of Cyclin E1 and stabilized its function to promote the cell proliferation. This mechanism and relationship reminded us that OTUB1/Cyclin E1 pathway might serve as a potential therapeutic target for PCa. When we interfere or interrupt the connection between OTUB1 and Cyclin E1, the proliferation and progression of PCa might be slowed or stopped. The results of RO-3306 treatment are the direct evidence of targeting OTUB1/Cyclin E1 axis for prostate cancer.

This new treatment may become an effective therapy for patients with PCa. The specific mechanism of OTUB1/Cyclin E1 axis needs further experiments to investigate and reinforce. Furthermore, the limitations of this research are also obvious, the role of Cyclin E1 in OTUB1 induced PC is not strong enough and it is only a correlation established from a mechanism. And the design of *in vivo* animal experiments was simple. The specific function of OTUB1/Cyclin E1 axis *in vivo* could not be fully explained by subcutaneous tumor related experiments. Although we have found that otub1 promoted the proliferation and progression of PCa via mediating and stabling Cyclin E1, in which one ubiquitination enzyme directly causing to the ubiquitination degradation of Cyclin E1 remains unclear, and the manner of OTUB1 regulating the expression of Cyclin E1 directly or indirectly are still yet not determined. Therefore, this research preliminarily proposed that OTUB1 could promote the progression and proliferation of PCa via regulating the expression of Cyclin E1, thus the specific internal mechanism will become the main work and direction in the next step.

## Conclusions

Here, our study shows that OTUB1 deubiquitinates and stabilizes Cyclin E1 to promote the progression, migration, and proliferation of prostate cancer. The OTUB1/Cyclin E1 axis may serve as a potential therapeutic target for patients with prostate cancer. The prognosis of patients with prostate cancer may be improved when the connection between OTUB1 and Cyclin E1 are interrupted or disturbed.

## Data Availability Statement

The datasets presented in this study can be found in online repositories. The names of the repository/repositories and accession number(s) can be found in the article/Supplementary Material.

## Ethics Statement

The animal study was reviewed and approved by Ethics Committee of the Second Hospital of Tianjin Medical University.

## Author Contributions

YL: project development, perform experiment, data analysis, and manuscript writing. NW: project development. KW: perform experiment. MW: data analysis. YW: perform experiment. JG: data collection. BZ: data collection. FM: project development. YW: data analysis. NJ: project development. All authors contributed to the article and approved the submitted version.

## Conflict of Interest

The authors declare that the research was conducted in the absence of any commercial or financial relationships that could be construed as a potential conflict of interest.

## Abbreviations

OTUB1: ovarian tumor domain deubiquitination 1
PCa: prostate cancer
CCNE1: Cyclin E1
AR: Androgen receptor
BPH: Benign prostate hyperplasia
IP: Immunoprecipitation
UB: Ubiquitination
DUB: Deubiquitination
ADT: androgen deprivation therapy
CRPC: castration-resistant prostate cancer
mCRPC: metastasis castration resistant prostate cancer.
