# Supplementary material for: OTUB1 Promotes Progression and Proliferation of Prostate Cancer via Deubiquitinating and Stabling Cyclin E1
Source: Front Cell Dev Biol. 2021 Jan 18;8:617758. doi: 10.3389/fcell.2020.617758 (PMC7848094; doi:10.3389/fcell.2020.617758)
Supplement: Supplementary file 1 [file Table_1.DOCX]

| Name | primers |
| --- | --- |
| Si control | UUCUCCGAACGUGUCACGUTT |
| Si otub1 #1 | CUCCGAAGGUGUUAACUGUTT |
| Si otub1 #2 | GACAACAUCUAUCAACAGATT |
| Si ccne1 #1 | GUUGCAUAUCUAAAUGACUTT |
| Si ccne1 #2 | UUCUCCGAACGUGUCACGUTT |

Table 1.
